# Predictive modeling of acute radiation-induced dermatitis in nasopharyngeal carcinoma patients undergoing tomotherapy using machine learning with multimodal data integration

**DOI:** 10.3389/fonc.2025.1601493

**Published:** 2025-10-02

**Authors:** Jiabiao Hong, Yuhao Lin, Xiaoting Lin, Linghui Yan, Jihong Chen, Huabing Chen, Miaomiao Zeng, Shuzhen Yuan

**Affiliations:** ^1^ Department of Radiation Oncology, Clinical Oncology School of Fujian Medical University, Fujian Cancer Hospital, Fuzhou, Fujian, China; ^2^ The First Affiliated Hospital, Hengyang Medical School, University of South China, Hengyang, Hunan, China; ^3^ Department of Sport Medicine, Southern Hospital Ganzhou Hospital (Ganzhou People’s Hospital), Ganzhou, Jiangxi, China

**Keywords:** acute radiation-induced dermatitis, predictive modeling, nasopharyngeal carcinoma, tomotherapy, machine learning

## Abstract

**Purpose:**

Radiation dermatitis (RD) is a common and debilitating side effect of radiotherapy in nasopharyngeal carcinoma (NPC) patients. Traditional predictive models lack sufficient accuracy for assessing acute radiation dermatitis (ARD) after tomotherapy treatment. This study aims to integrate clinical, dosimetric, and radiomic features to enhance the accuracy and robustness of predictions, thereby promoting a more personalized risk assessment for NPC patients undergoing tomotherapy.

**Methods:**

A cohort of 161 NPC patients who underwent Tomotherapy was retrospectively analyzed. Clinical, dosimetric, and radiomic features were extracted for the purpose of model development. Feature selection was conducted using statistical tests and Least Absolute Shrinkage and Selection Operator(LASSO) regression. Several machine learning algorithms were then employed to construct the predictive models, including Logistic Regression, Support Vector Machine (SVM), K-Nearest Neighbors (KNN), Random Forest, Extra Trees, XGBoost, Light Gradient Boosting Machine (LightGBM), and Multilayer Perceptron (MLP). These models were built based on clinical, radiomic, dosiomic, and combined feature sets. Model performance was assessed by evaluating the area under the receiver operating characteristic curve (AUC), sensitivity, and specificity. To ensure fairness in comparisons, five-fold cross-validation was applied during the training of all models in the training cohort.

**Results:**

The combined model, integrating clinical, radiomic, and dosiomic features, demonstrated the highest predictive accuracy, achieving an AUC of 0.916 (95% CI: 0.866–0.967) in the training cohort and 0.797 (95% CI: 0.616–0.978) in the validation cohort. In comparison, the clinical model (AUC=0.704), radiomic model (AUC=0.865), and dosiomic model (AUC=0.640) had lower predictive performance. SVM method demonstrated superior overall performance across various model constructions. The combined model based on the SVM method exhibited optimal predictive performance, achieving the best results in both the test and validation cohorts.

**Conclusions:**

The developed combined prediction system achieves superior performance in anticipating severe ARD in NPC undergoing tomotherapy cases. This tool facilitates pre-therapeutic risk stratification, dosimetric parameter refinement, and evidence-based scheduling of preventive skin management protocols, offering a paradigm-shifting approach to individualized cutaneous protection strategies.

## Introduction

Radiotherapy (RT) is used as a standard treatment modality for nasopharyngeal carcinoma (NPC). To ensure treatment efficacy and minimize damage to normal tissues, RT continues to evolve and develop through the efforts of radiation oncologists (1). In the early 21st century, intensity-modulated radiotherapy(IMRT), using dynamic multi-leaf collimators and inverse planning algorithms, achieved precise dose sculpting for complex tumor shapes (2), upon which tomotherapy emerged as a more advanced form of conformal RT. It combines continuous helical IMRT delivery with high-precision Image guided radiotherapy(IGRT) through integrated computer tomography(CT) scanning (3). Particularly in the treatment field of NPC, tomotherapy minimizes the risk of damage to normal tissues during the treatment process of NPC patients, ensuring patient safety and treatment efficacy.

Nevertheless, due to inherent limitations in radiation dose distribution, adjacent healthy tissues inevitably receive radiation exposure, causing complications such as dermatitis, xerostomia, and dysphagia. However, during the radiotherapy process for NPC, various normal organs inevitably receive radiation exposure, resulting in numerous adverse reactions, such as dermatitis, xerostomia, dysphagia, hypopituitarism, and lower cranial nerve complications. Among these, acute radiation dermatitis (ARD) is common, with incidences of grade ≥3 dermatitis ranging from 0% to 44% among patients receiving tomotherapy, depending on treatment and patient-specific risk factors (4–7). Radiation dermatitis (RD) are classified into acute and chronic types, including acute reactions characterized by erythema, dry and moist desquamation, pruritus, bleeding, ulceration, and skin infections, as well as chronic changes such as chronic atrophy and fibrosis. Moreover, severe radiation dermatitis may require radiation dose limitation or even treatment discontinuation (8). Therefore, the prevention and management of RD remains a challenge.

Analyzing and predicting RD for early intervention through data analysis and modeling is one of the directions pursued by clinicians. However, current research primarily focuses on prediction using dose parameters from Dose and Volume Histogram(DVH) and clinical factors (5, 6), with limited accuracy of these methods. For example, Lee et al (9). reported an AUC of just 0.62 for a machine-learning model that used clinical and DVH variables in a breast-cancer cohort, and Bonomo et al (10). showed similarly limited predictive performance when DVH-based skin-dose metrics were applied to head-and-neck squamous-cell carcinoma. Meanwhile, there is also a lack of prediction models suitable for patients receiving tomo radiotherapy. In recent years, emerging radiomics and dosomics have provided new directions for the diagnosis and prediction of tumors and complications. Radiomics is an emerging effective method for quantitative analysis of radiological images (11, 12). By mining high-dimensional features from imaging data, it not only plays an important role in risk stratification and differential diagnosis, but also shows great potential in prognosis prediction, treatment sensitivity assessment, and early identification of related complications (13–15). Dosomics, through examination of dose distribution uniformity and spatial variability, provides a deeper understanding than DVH.

Therefore, this study aims to develop a robust predictive model combining clinical parameters, dosimetric information, and CT-based radiomics features, thereby providing personalized risk assessment for ARD in NPC patients undergoing tomotherapy.

## Materials and methods

### Patients

This study included 161 nasopharyngeal carcinoma (NPC) patients who received complete treatment at Fujian Cancer Hospital between January 2023 and January 2024. Patients were stratified by outcome and then randomly split into a training set and a validation set in an 8:2 ratio. Eligible patients met all of the following inclusion criteria : (1) newly diagnosed, pathologically confirmed nasopharyngeal carcinoma scheduled for radical tomotherapy; (2) age 18–70 years to minimize age-related confounding; (3) no pre-existing dermatological conditions likely to confound radiation-induced skin reactions, including chronic inflammatory dermatoses (e.g., eczema, psoriasis), connective-tissue or immune-mediated disorders (e.g., scleroderma), chronic ulcers or infections within the planned irradiation field, or pre-existing chronic skin damage; (4) an ECOG performance status of 0–1 indicating fitness for full-dose treatment and none of the exclusion criteria: (1) prior head-and-neck radiotherapy or neck surgery, which would distort dose distribution and healing; (2) a history of other malignancies, whose treatments could influence skin toxicity; (3) severe cardiovascular or systemic disease likely to require dose modification or interruption and independently affect skin reactions; and (4) incomplete follow-up data for radiation-dermatitis assessment, which would compromise outcome validity.

### Treatment and ARD evaluation

All patients were staged according to the 8th edition of the Union for International Cancer Control(UICC)/American Joint Commission on Cancer(AJCC) staging system and treatment plans were determined, with stage I patients receiving radical radiotherapy, stage II patients receiving combined chemoradiotherapy, and stage III-IVB patients receiving radiotherapy and other combination treatments. According to our center’s previously reported target - volume delineation criteria, experienced radiation oncologists with over 5 years of experience delineated the gross tumor volume(GTV), clinical target volume(CTV), planning target volume(PTV), and organs at risk(OARs) regions (16). The prescribed radiotherapy doses were as follows: GTV: 70-72.6 Gy/31–33 fractions, CTV1: 62-62.7 Gy/31–33 fractions, CTV2: 54.4-56.2 Gy/31–33 fractions. All patients received IMRT using the Accuray TomoHD helical tomotherapy system (Accuray Inc., Madison, Wisconsin). The radiation energy used was 6 MV, with a dose rate of 850 MU/min, and the dose calculation algorithm employed was the convolution/superposition (C/S) algorithm within the treatment planning system. The voxel spatial resolution for dose calculation was 0.273 × 0.273 × 0.3 cm^3^.

After treatment initiation, all patients underwent clinical assessment weekly for ARD by experienced radiation oncologists according to Radiation Therapy Oncology Group(RTOG) scoring criteria (17), with grade ≥III considered severe ARD.

### Image acquisition and contour delineation

**Figure 1:**
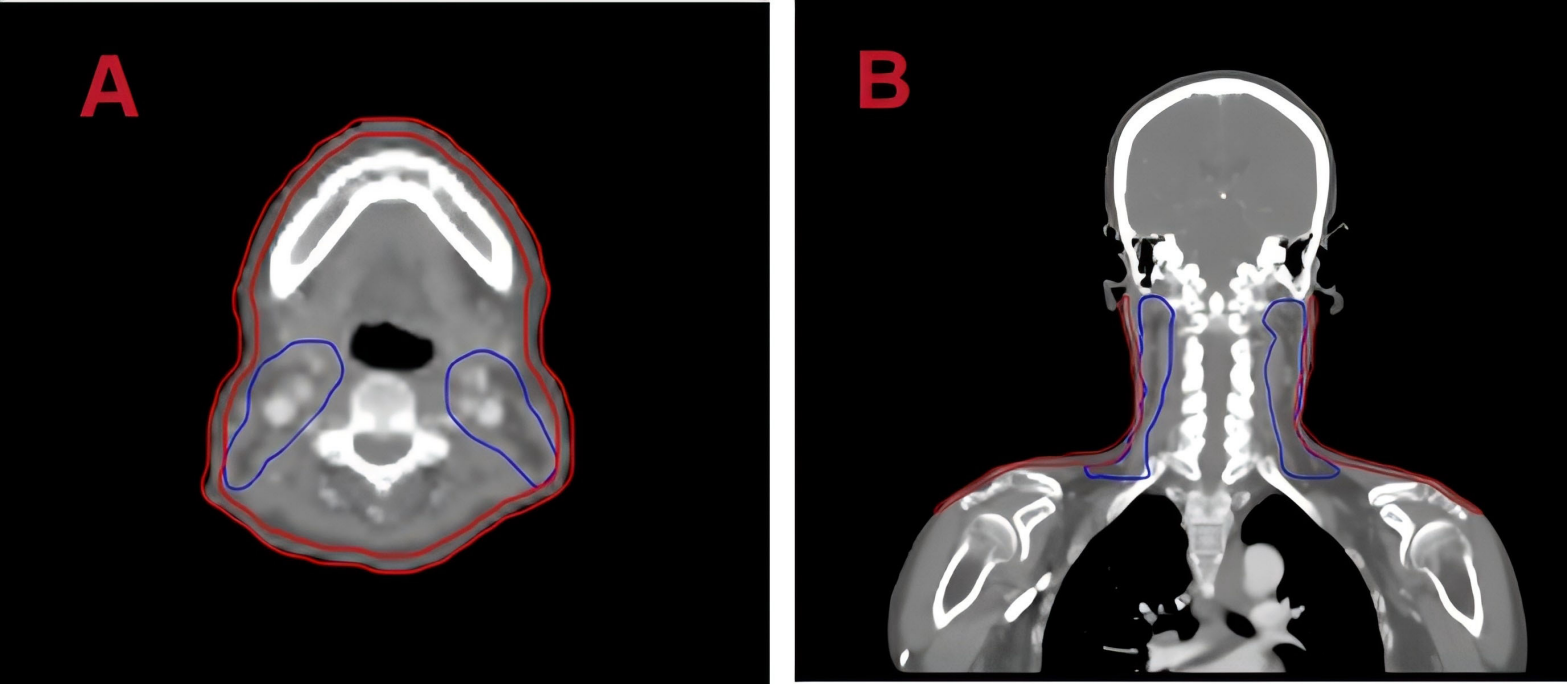
Regions of interest (ROI) on planning CT images. **(A)** Axial and **(B)** coronal views with skin delineated in red.

Positional CT scans were performed using a Philips Brilliance large-bore CT scanner. Patients were immobilized in the supine position using thermoplastic masks and customized foam. The tube voltage was set to 120 kV, X-ray tube current was 225 mA, CT slice thickness was 3mm, and scan resolution was 512 × 512 pixels. To analyze the dose distribution in the patient’s superficial skin layer, ring - shaped structures were automatically generated as regions of interest(ROIs) by subtracting 3 millimeters from the patient’s surface. The upper and lower boundaries of these ROIs were consistent with the upper and lower boundaries of the planning target volume for the lymph nodes (PTV-ND), as shown in the delineated ROI figure (**Figure 1**).

### Radiomic and dosiomic features extracted

In this study, radiomic and dosiomic features were extracted from the ROIs using the open-source Pyradiomics package based on the Python 3.7 platform. The extracted radiomic features were categorized into three groups: first-order statistical features, shape features, and texture features. First-order statistical features represent variations in symmetry, uniformity, and local intensity distributions within the measured ROI. Shape features provide a quantitative description of the three-dimensional size and morphology of the ROIs. Texture features reflect the spatial arrangement of the grayscale values within the ROIs. For detailed descriptions of each feature type, please refer to the official Pyradiomics documentation (18). To prevent large differences in the range of variable values, Z-score standardization of features was used to improve model convergence speed and coefficient comparability.

### Clinical features

A total of 25 clinical features were included, comprising age, gender, T stage, N stage, overall staging, the season during treatment, pre-treatment Body Mass Index(BMI), smoking, alcohol consumption, diabetes, hypertension, thyroid diseases, cervical lymph nodes, radiotherapy sensitizing drug, induction chemotherapy, concurrent chemotherapy, concurrent targeted therapy, immunotherapy, use of hormonal drugs, pre-treatment total protein, pre-treatment albumin, pre-treatment white blood cell count, pre-treatment platelet count, pre-treatment hemoglobin, and pre-treatment neutrophils.

### Feature selection and construction of model

Univariate logistic regression was first performed to identify clinical variables significantly associated with the outcome. Variables with p < 0.05 in univariate analysis were subsequently entered into a multivariate logistic regression model to adjust for potential confounding factors and to determine independent predictors.

Radiomic and dosiomic features were screened by contrasting patients who developed ARD with those who did not. The Kolmogorov–Smirnov test was first applied to assess the normality of each feature’s distribution. Features with p-values below 0.05 were considered non-normally distributed, while those with p-values above this threshold were assumed to follow a normal distribution. Based on these results, normally distributed features were compared between groups using Student’s t-test, and non-normally distributed features were analyzed with the Mann–Whitney U test. For both tests, the null hypothesis assumed no significant difference between groups for a given feature. Features with p-values > 0.05 were considered non-discriminative and, if appearing in pairs of highly similar features, one was randomly excluded to reduce redundancy. Additionally, we used the Spearman rank correlation coefficient to measure the correlation between highly related features. When the correlation coefficient between two features exceeded 0.9, a greedy recursive elimination strategy was adopted to retain only one of these features, discarding the feature with the highest average correlation at each iteration. The Least Absolute Shrinkage and Selection Operator (LASSO) regression model was applied to the radiomic and dosiomic feature sets to select relevant variables for constructing the respective radiomic and dosiomic models. For the combined model, all candidate clinical variables (without prior filtering), together with radiomic and dosiomic features, were pooled and subjected to LASSO regression for feature selection, enabling early fusion of all feature types before model training.

Finally, the selected features were employed to construct four types of risk models: clinical, radiomic, dosiomic, and combined, using a range of machine learning algorithms, including Logistic Regression, Support Vector Machine, K-Nearest Neighbors, Random Forest, Extra Trees, XGBoost, LightGBM, and Multilayer Perceptron. For each model type, the algorithm with the best overall performance—considering both training and validation results—was selected as the final predictive model. To ensure fairness in comparisons, five-fold cross-validation was applied during the training of all models in the training cohort.

### Statistical analysis

To examine the equivalence of patient characteristics across different cohorts, normally distributed continuous data were analyzed using independent t-tests, whereas non-normally distributed data (expressed as medians with interquartile ranges) were analyzed with Mann-Whitney U tests. Categorical variables were analyzed using chi-square tests. Furthermore, we evaluated the predictive performance of the four models using receiver operating characteristic(ROC) curves, from which we calculated the area under the ROC curve(AUC) and the balance between sensitivity and specificity at the maximum Youden index. Additionally, we assessed the performance of all four models in both the training and testing cohorts and used decision curve analysis (DCA) to evaluate the clinical utility of the combined model. Analyses were conducted using SPSS (version 21.0; IBM Corporation) and the Pytorch 1.8.0-based “One-Click AI” platform (http://www.medai.icu). A two-sided p-value of ≤ 0.05 was considered statistically significant.

## Result

### Patient characteristics

Baseline characteristics of patients in the training and test cohorts are summarized in **Table 1**. The mean age of patients in the training cohort was 45.90 ± 11.31 years, including 125 males (70.54%) and 50 females (29.46%). The mean age of patients in the test cohort was 46.38 ± 10.65 years, including 35 males (68.75%) and 9 females (31.25%). Severe ARD occurred in 44 patients (34.11%) in the training cohort and 11 patients (34.38%) in the validation cohort.

**Table 1:** Baseline characteristics of patients in the training and test cohorts.

Feature	Train cohort				Test cohort			
	[ALL]	ARD<III	ARD≥III	P-value	[ALL]	ARD<III	ARD≥III	P-value
	*N=129*	*N=85*	*N=44*		*N=32*	*N=21*	*N=11*	
Sex				0.069				0.452
Male	91(70.54)	55(64.71)	36(81.82)		22(68.75)	13(61.90)	9(81.82)	
Female	38(29.46)	30(35.29)	8(18.18)		10(31.25)	8(38.10)	2(18.18)	
Age	45.90 ± 11.31	45.67 ± 11.19	46.34 ± 11.66	0.751	46.38 ± 10.65	45.95 ± 12.62	47.18 ± 5.67	0.762
Age Grade				0.916				0.269
18-44	59(45.74)	40(47.06)	19(43.18)		11(34.38)	8(38.10)	3(27.27)	
45-59	56(43.41)	36(42.35)	20(45.45)		18(56.25)	10(47.62)	8(72.73)	
≥60	14(10.85)	9(10.59)	5(11.36)		3(9.38)	3(14.29)	null	
Weight	67.65 ± 13.74	64.95 ± 12.49	72.87 ± 14.67	0.001	67.79 ± 12.13	63.78 ± 9.95	75.45 ± 12.64	0.007
BMI				0.043				0.056
≤18.5	3(2.33)	3(3.53)	null		0(0.00)	null	null	
18.5-23.9	60(46.51)	46(54.12)	14(31.82)		16(50.00)	13(61.90)	3(27.27)	
24.0-27.9	51(39.53)	27(31.76)	24(54.55)		13(40.62)	8(38.10)	5(45.45)	
28.0-32.0	12(9.30)	8(9.41)	4(9.09)		2(6.25)	null	2(18.18)	
>32.0	3(2.33)	1(1.18)	2(4.55)		1(3.12)	null	1(9.09)	
T category				0.093				0.199
T0	2(1.55)	2(2.35)	null		0(0.00)	null	null	
T1	24(18.60)	18(21.18)	6(13.64)		6(18.75)	2(9.52)	4(36.36)	
T2	15(11.63)	11(12.94)	4(9.09)		5(15.62)	4(19.05)	1(9.09)	
T3	70(54.26)	39(45.88)	31(70.45)		11(34.38)	9(42.86)	2(18.18)	
T4	18(13.95)	15(17.65)	3(6.82)		10(31.25)	6(28.57)	4(36.36)	
N category				0.369				0.620
N0	13(10.08)	8(9.41)	5(11.36)		3(9.38)	2(9.52)	1(9.09)	
N1	49(37.98)	34(40.00)	15(34.09)		13(40.62)	9(42.86)	4(36.36)	
N2	34(26.36)	25(29.41)	9(20.45)		11(34.38)	8(38.10)	3(27.27)	
N3	33(25.58)	18(21.18)	15(34.09)		5(15.62)	2(9.52)	3(27.27)	
M category				0.246				1.000
M0	124(96.12)	80(94.12)	44(100.00)		32(100.00)	21(100.00)	11(100.00)	
M1	5(3.88)	5(5.88)	null		0(0.00)	null	null	
Stage				0.154				0.148
I	10(7.75)	9(10.59)	1(2.27)		4(12.50)	4(19.05)	null	
II	68(52.71)	42(49.41)	26(59.09)		14(43.75)	10(47.62)	4(36.36)	
III	47(36.43)	30(35.29)	17(38.64)		14(43.75)	7(33.33)	7(63.64)	
IV	4(3.10)	4(4.71)	null		0(0.00)	null	null	
Cervical Lymph Node Metastasis				0.968				1.000
No	13(10.08)	8(9.41)	5(11.36)		3(9.38)	2(9.52)	1(9.09)	
Yes	116(89.92)	77(90.59)	39(88.64)		29(90.62)	19(90.48)	10(90.91)	
Treatment Time (season)				0.414				0.306
Spring	70(54.26)	47(55.29)	23(52.27)		19(59.38)	14(66.67)	5(45.45)	
Summer	51(39.53)	31(36.47)	20(45.45)		12(37.50)	6(28.57)	6(54.55)	
Autumn	4(3.10)	4(4.71)	null		1(3.12)	1(4.76)	null	
Western	4(3.10)	3(3.53)	1(2.27)		0(0.00)	null	null	
Radiotherapy Sensitizer				0.196				1.000
No	39(30.23)	22(25.88)	17(38.64)		8(25.00)	5(23.81)	3(27.27)	
Yes	90(69.77)	63(74.12)	27(61.36)		24(75.00)	16(76.19)	8(72.73)	
Induction chemotherapy				0.344				1.000
No	8(6.20)	7(8.24)	1(2.27)		3(9.38)	2(9.52)	1(9.09)	
Yes	121(93.80)	78(91.76)	43(97.73)		29(90.62)	19(90.48)	10(90.91)	
Concurrent chemotherapy				0.640				0.625
No	77(59.69)	49(57.65)	28(63.64)		17(53.12)	10(47.62)	7(63.64)	
Yes	52(40.31)	36(42.35)	16(36.36)		15(46.88)	11(52.38)	4(36.36)	
Targeted Therapy				0.564				1.000
No	13(10.08)	10(11.76)	3(6.82)		3(9.38)	2(9.52)	1(9.09)	
Yes	116(89.92)	75(88.24)	41(93.18)		29(90.62)	19(90.48)	10(90.91)	
Immunotherapy				0.466				1.000
No	111(86.05)	75(88.24)	36(81.82)		29(90.62)	19(90.48)	10(90.91)	
Yes	18(13.95)	10(11.76)	8(18.18)		3(9.38)	2(9.52)	1(9.09)	
Hormone Drugs				0.329				1.000
No	47(36.43)	34(40.00)	13(29.55)		5(15.62)	3(14.29)	2(18.18)	
Yes	82(63.57)	51(60.00)	31(70.45)		27(84.38)	18(85.71)	9(81.82)	
Smoking				0.968				0.423
No	116(89.92)	77(90.59)	39(88.64)		27(84.38)	19(90.48)	8(72.73)	
Yes	13(10.08)	8(9.41)	5(11.36)		5(15.62)	2(9.52)	3(27.27)	
Drinking				0.553				1.000
No	121(93.80)	81(95.29)	40(90.91)		30(93.75)	20(95.24)	10(90.91)	
Yes	8(6.20)	4(4.71)	4(9.09)		2(6.25)	1(4.76)	1(9.09)	
Thyroid Disease				0.173				1.000
No	123(95.35)	79(92.94)	44(100.00)		29(90.62)	19(90.48)	10(90.91)	
Yes	6(4.65)	6(7.06)	null		3(9.38)	2(9.52)	1(9.09)	
Diabetes				1.000				1.000
No	128(99.22)	84(98.82)	44(100.00)		32(100.00)	21(100.00)	11(100.00)	
Yes	1(0.78)	1(1.18)	null		0(0.00)	null	null	
Hypertension				1.000				0.933
No	114(88.37)	75(88.24)	39(88.64)		25(78.12)	17(80.95)	8(72.73)	
Yes	15(11.63)	10(11.76)	5(11.36)		7(21.88)	4(19.05)	3(27.27)	
Pathology				0.927				1.000
No	7(5.43)	4(4.71)	3(6.82)		3(9.38)	2(9.52)	1(9.09)	
Yes	122(94.57)	81(95.29)	41(93.18)		29(90.62)	19(90.48)	10(90.91)	
Total Protein				0.233				1.000
Normal	116(89.92)	74(87.06)	42(95.45)		28(87.50)	18(85.71)	10(90.91)	
Abnormal	13(10.08)	11(12.94)	2(4.55)		4(12.50)	3(14.29)	1(9.09)	
Albumin				1.000				0.415
Normal	93(72.09)	61(71.76)	32(72.73)		25(78.12)	15(71.43)	10(90.91)	
Abnormal	36(27.91)	24(28.24)	12(27.27)		7(21.88)	6(28.57)	1(9.09)	
Leukocyte				0.422				1.000
Normal	95(73.64)	65(76.47)	30(68.18)		20(62.50)	13(61.90)	7(63.64)	
Abnormal	34(26.36)	20(23.53)	14(31.82)		12(37.50)	8(38.10)	4(36.36)	
Platelet				0.196				0.592
Normal	90(69.77)	63(74.12)	27(61.36)		26(81.25)	16(76.19)	10(90.91)	
Abnormal	39(30.23)	22(25.88)	17(38.64)		6(18.75)	5(23.81)	1(9.09)	
Hemoglobin				0.446				0.068
Normal	30(23.26)	22(25.88)	8(18.18)		12(37.50)	5(23.81)	7(63.64)	
Abnormal	99(76.74)	63(74.12)	36(81.82)		20(62.50)	16(76.19)	4(36.36)	
Neutrophile Granulocyte				0.149				0.315
Normal	91(70.54)	64(75.29)	27(61.36)		21(65.62)	12(57.14)	9(81.82)	
Abnormal	38(29.46)	21(24.71)	17(38.64)		11(34.38)	9(42.86)	2(18.18)	

Initialism; ARD, acute radiation dermatitis; BMI, Body Mass Index.

### Construction of clinical, radiomics and dosiomics model

Following feature selection through univariate and multivariate regression analyses, two significant clinical variables (weight and BMI) were identified. The clinical model using LightGBM demonstrated good predictive performance, with an AUC of 0.704 (95% CI, 0.613-0.786) in the training cohort and 0.753 (95% CI, 0.580-0.926) in the validation cohort (**Supplementary Table 1**).

A total of 1835 radiomic features were extracted from each patient. Following feature selection, 14 features with non-zero coefficients were retained for model construction. The constructed radiomics model exhibited optimal performance using SVM, outperforming other machine learning approaches. In the training cohort, the model demonstrated an AUC of 0.865 (95% CI 0.795-0.935) with sensitivity and specificity values of 0.932 and 0.671. External validation achieved an AUC of 0.779 (95% CI 0.604-0.954) with sensitivity and specificity values of 0.727 and 0.762, respectively (**Supplementary Table 2**).

107 dosomic features were extracted from each patient. Consistent with the radiomics approach, the dosomics model employed analogous feature filtering methodology. A single predictive feature survived selection and was incorporated into the final dosomics model. The logistic regression model demonstrated optimal performance, achieving a training AUC of 0.640 (95% CI 0.540-0.740) with sensitivity and specificity values of 0.795 and 0.459, respectively. External validation yielded comparable performance (AUC=0.641, 95% CI 0.421-0.861) with sensitivity and specificity values of 0.545 and 0.741, respectively (**Supplementary Table 3**).

### Construction of combined model

**Figure 2:**
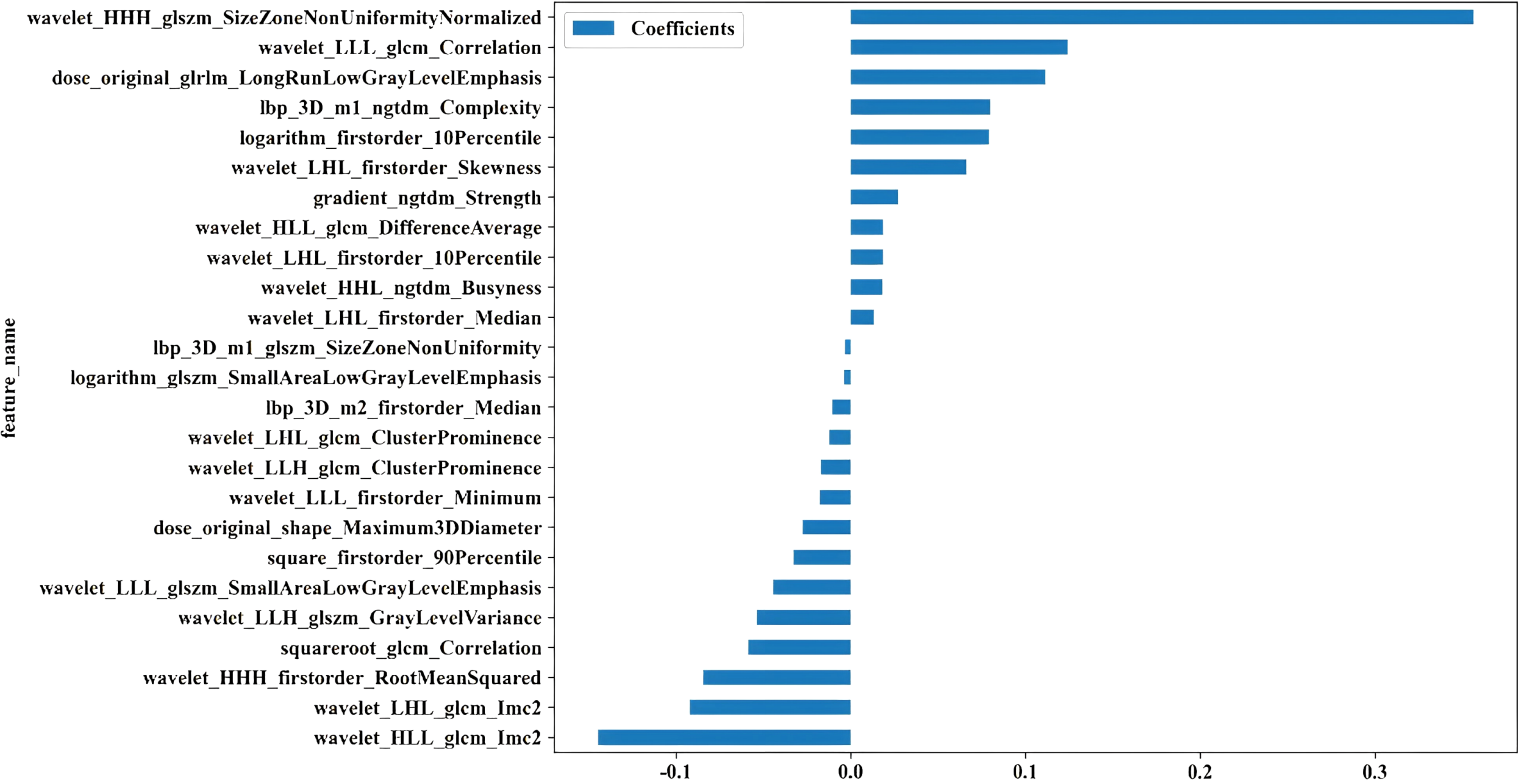
Coefficient distributions for the 25 most informative predictors in the combined model.

Twenty-five features were identified as the most informative predictors in the combined model, with their coefficient distributions and importance rankings visualized in **Figure 2**. Among all evaluated algorithms, the SVM-based model demonstrated superior predictive performance. In the training cohort, the model achieved an exceptional AUC of 0.916 (95% CI 0.866-0.967) with sensitivity and specificity values of 0.932 and 0.765. External validation yielded an AUC of 0.797 (95% CI 0.616-0.978) with sensitivity and specificity values of 0.727 and 0.762, respectively (**Table 2**).

**Table 2:** Predictive performance of all combined models.

	Accuracy	AUC	95%Cl	Sensitivity	Specificity	PPV	NPV	Precision	Recall	F1	Threshold
LR											
Train Cohort	0.760	0.888	0.8317-0.9443	0.955	0.659	0.592	0.966	0.592	0.955	0.730	0.253
Test Cohort	0.781	0.792	0.6131-0.9713	0.636	0.857	0.700	0.818	0.700	0.636	0.667	0.406
SVM											
Train Cohort	0.822	0.916	0.8655-0.9666	0.932	0.765	0.672	0.956	0.672	0.932	0.781	0.299
Test Cohort	0.750	0.797	0.6155-0.9776	0.727	0.762	0.615	0.842	615.000	0.727	0.667	0.342
KNN											
Train Cohort	0.729	0.794	0.7196-0.8687	0.432	0.882	0.655	0.750	0.655	0.432	0.521	0.400
Test Cohort	0.688	0.710	0.5230-0.8969	0.273	0.905	0.600	0.704	0.600	0.273	0.375	0.400
RandomForest											
Train Cohort	0.814	0.922	0.8735-0.9709	0.909	0.765	0.667	0.942	0.667	0.909	0.769	0.313
Test Cohort	0.781	0.823	0.6478-0.9972	0.727	0.810	0.667	0.850	0.667	0.727	0.696	0.375
ExtraTrees											
Train Cohort	0.767	0.838	0.7690-0.9064	0.727	0.788	0.640	0.848	0.640	0.727	0.681	0.362
Test Cohort	0.688	0.623	0.3946-0.8521	0.545	0.762	0.545	0.762	545.000	0.545	0.545	0.359
XGBoost											
Train Cohort	0.977	0.995	0.9863-1.0000	0.955	0.988	0.977	0.977	0.977	0.955	0.966	0.456
Test Cohort	0.562	0.662	0.4701-0.8546	0.909	0.381	0.435	0.889	0.435	0.909	0.588	0.131
LightGBM											
Train Cohort	0.853	0.897	0.8429-0.9515	0.773	0.894	0.791	0.884	0.791	0.773	0.782	0.398
Test Cohort	0.531	0.675	0.4806-0.8700	0.909	0.333	0.417	0.875	0.417	0.909	0.571	0.223
MLP											
Train Cohort	0.659	0.802	0.7266-0.8771	0.932	0.518	0.500	0.936	0.500	0.932	0.651	0.298
Test Cohort	0.688	0.719	0.5076-0.9297	0.636	0.714	0.538	0.789	0.538	0.636	0.583	0.348

AUC, area under the receiver operating characteristic curve; KNN, K-Nearest Neighbors; LightGBM, Light Gradient Boosting Machine; LR, Logistic Regression; MLP, Multilayer Perceptron; NPV, Negative Predictive Value; PPV, Positive Predictive Value; SVM, Support Vector Machine; XGboost, eXtreme Gradient Boosting.

### Validating model performance

**Figure 3:**
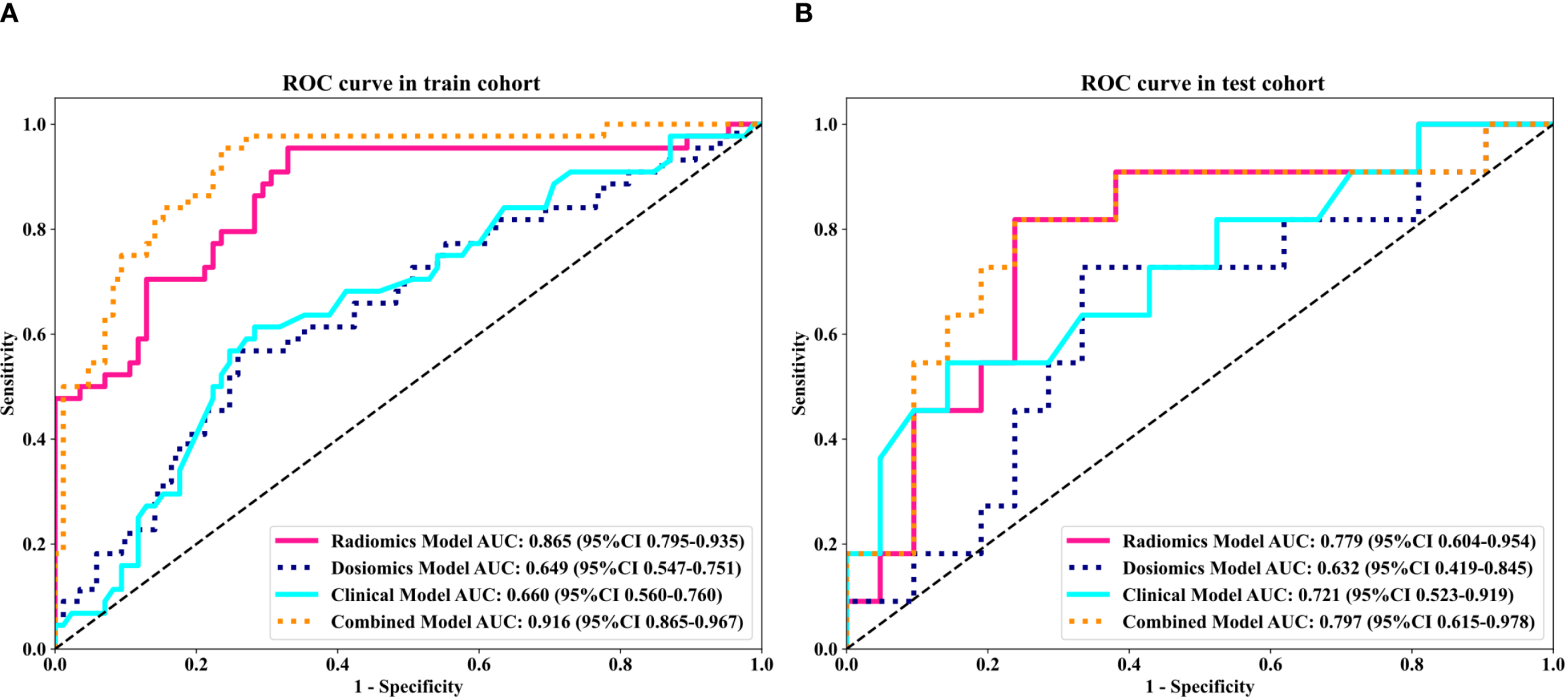
Comparison of receiver operating characteristic (ROC) curves for the clinical, dosomic, radiomics, and combined models.

**Figure 4:**
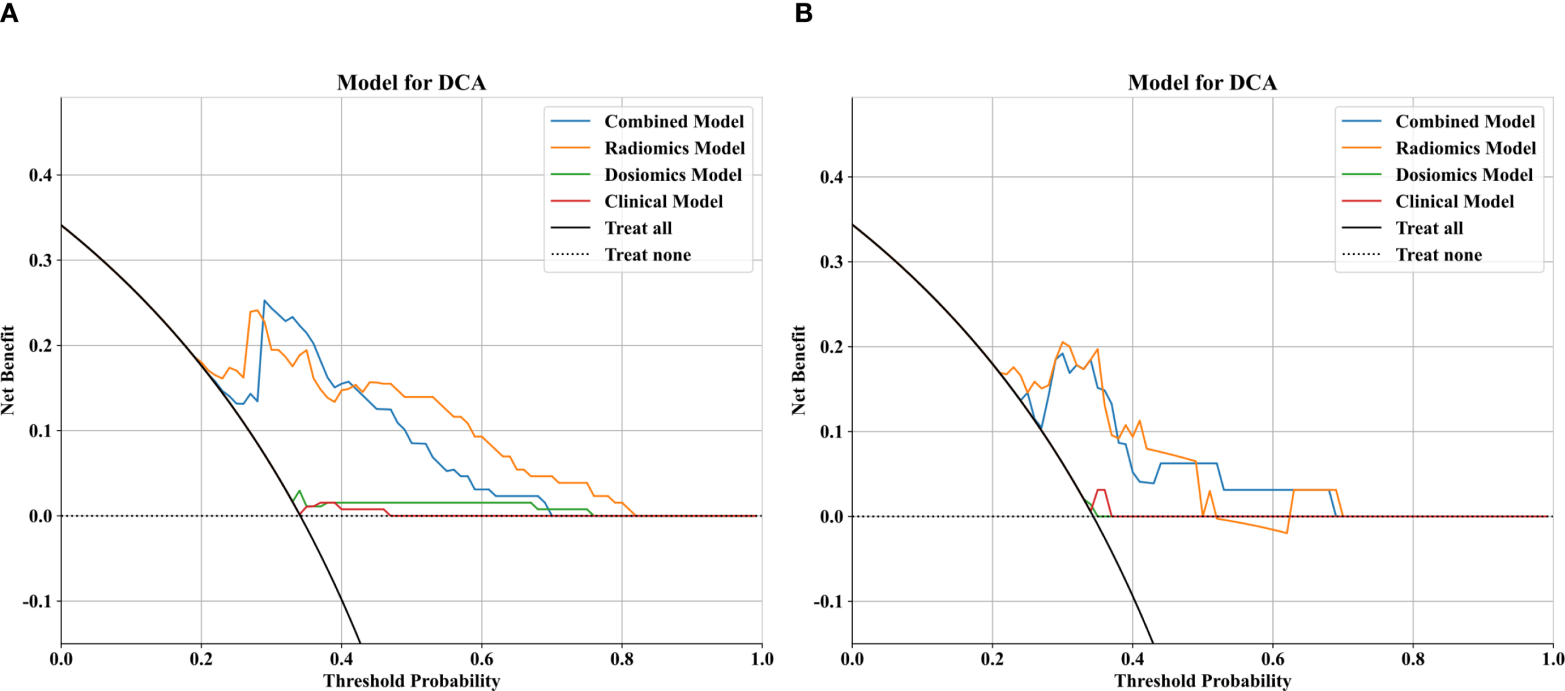
Comparison of decision curve analysis (DCA) curves for the clinical, dosomic, radiomics, and combined models.

To further analyze the predictive performance of the models, we selected the SVM method, which demonstrated superior overall performance in various model constructions, for comparison. The ROC curves showed that the combined model exhibited optimal predictive performance in both test and validation cohorts in **Figure 3**. Additionally, DCA revealed that across all cohorts, within a threshold probability range of 20%-75%, the combined model showed similar net benefits to the radiomics model but significantly outperformed other models in terms of net benefits, enhancing clinical utility (**Figure 4**).

## Discussion

To address the gap in existing research on ARD prediction for NPC patients undergoing tomotherapy, we developed a novel integrated model combining clinical parameters, radiomic features, and dosomic features using multiple machine learning methods. Our results show that the optimal combined model achieved AUC values of 0.916 in the training cohort and 0.797 in the test cohort, outperforming single-feature models in terms of discriminative ability, goodness-of-fit, and diagnostic performance. This study highlights the potential of radiomics and dosomics approaches in predicting severe ARD reactions in NPC patients receiving tomotherapy, confirming their capacity to provide broader evaluations and contributions for assessing ARD severity.

ARD is a frequent complication in NPC radiotherapy, primarily due to the common co-occurrence of cervical lymph node metastasis at initial diagnosis. Although advancements in radiation techniques have been made, the inherent physical properties of radiotherapy, such as the rapid dose fall-off outside the treatment field, still result in collateral radiation exposure to surrounding healthy tissues, leading to skin radiation effects. Our total experimental cohort included 161 patients, with a severe ARD incidence (≥ grade 3) of 34.16%. In line with previous research, the incidence of severe ARD shows significant variability (4–6). These differences are likely due to variations in cohort characteristics and treatment protocols across studies, including tumor volume, tumor-to-skin distance, radiation techniques, and concurrent chemoradiotherapy (19).

The occurrence and severity of radiation dermatitis are influenced by both patient-specific risk factors and treatment protocols. Structural uniformity and tissue consistency within treatment fields serve as pivotal factors modulating radiation dermatitis pathogenesis. The presence of skin folds in treatment zones correlates with enhanced cutaneous reactions due to intertriginous contact promoting moisture retention, elevated temperatures, and frictional forces (20). BMI, reflecting skin fold-related parameters, is frequently employed as a clinical predictor for severe acute dermatological toxicity (21, 22). In our analysis, body weight and BMI were identified as critical predictive factors for ARD, corroborating previous studies. Treatment-associated variables encompass radiation modality, fraction size, total cumulative dose, and anatomical irradiation sites (23). Single radiation fractions exceeding 2 Gy are linked to exacerbated late-phase cutaneous effects (24), while concurrent chemotherapy intensifies cutaneous toxicity through synergistic mechanisms (25). Radiation dermatitis incidence also varies by anatomical site, with more severe reactions observed in facial, cervical, upper dorsal, and thoracic regions (21). These areas are difficult to assess quantitatively due to interobserver variability, subjective interpretations, and analytical fatigue, which can lead to critical data being overlooked. Radiomics and dosomics offer a robust solution by extracting wavelet-transformed features that capture subtle cellular damage and vascular changes, providing a more objective and reproducible assessment of tissue response to radiotherapy (26). These features elucidate subtle subclinical tissue modifications through scale- and orientation-dependent variations in high-/low-frequency signal components. High-frequency signals predominantly map microstructural perturbations (e.g., cellular injury, capillary remodeling), while low-frequency components characterize macroscopic anatomical patterns (27). These methods uncover high-dimensional tissue modifications, enhancing our ability to predict ARD development.

Current interventions for radiation dermatitis predominantly rely on clinician experience, anecdotal evidence, or low-grade studies, with critically limited prospectively validated data to guide therapeutic decision-making. Therapeutic objectives primarily focus on optimizing patient comfort, mitigating secondary injury risks, and accelerating wound re-epithelialization. Our predictive model stratifies ARD risk in NPC patients initiating radiotherapy, enabling personalized risk assessment, targeted patient education, and precision therapeutic planning. This framework can be embedded in clinical information systems, regardless of the specific radiotherapy technique, such as tomotherapy, VMAT, or other modern platforms, to create standardized cutaneous toxicity risk stratification protocols and ARD early alert tools. Early identification of high-risk cohorts through this system permits preemptive interventions that attenuate dermatitis severity, ultimately improving therapeutic outcomes, patient-reported quality metrics, and long-term clinical trajectories.

Our findings are consistent with a growing body of evidence that adding radiomic and dosiomic descriptors to conventional inputs meaningfully boosts skin-toxicity prediction. In our own data the LightGBM clinical-only model reached an AUC of 0.704 in training and 0.753 in external testing, whereas the early-fusion model that combined unfiltered clinical variables with LASSO-selected radiomic and dosiomic features improved performance to 0.916 and 0.797, respectively. A recent study (9) reported similar gains: integrating clinical, radiomic, and dosiomic features lifted the AUC from 0.62 to 0.83, compared with using only clinical and DVH variables in breast cancer. Li et al (28). reported that a random-forest model combining ten radiomic features, three dosimetric variables and six clinical factors achieved an AUC of 0.946 (95 % CI 0.887–0.987) in a breast cancer cohort. Xiang et al (29). demonstrated that a nomogram integrating DL, dosiomic features, and clinical factors achieved an AUC of 0.945, 0.916, and 0.832 in the training, internal, and external validation sets, respectively. Collectively, these findings reinforce the complementary value of the two modalities: radiomics contributes micro-textural information, and dosiomics captures spatial dose heterogeneity-signals that clinical and DVH variables alone cannot convey.

Nevertheless, this single-center retrospective study has inherent biases, and the limited sample size (n = 161), particularly the smaller test cohort (n = 33) relative to the training cohort (n = 129), may affect model stability and generalizability. Future multi-center studies and external validation using heterogeneous datasets acquired from different scanners and imaging protocols are needed to confirm the robustness of the model in diverse clinical settings. Furthermore, our ARD grading relied on clinicians’ empirical assessments, while variability in patient adherence to skin care protocols may introduce confounding effects. Finally, the mechanistic underpinnings connecting radiomic signatures to histopathological changes remain unvalidated, necessitating cautious interpretation of our findings. Future investigations should incorporate histopathological and genomic biomarkers to develop multidimensional predictive frameworks, ensuring biological plausibility while advancing personalized therapeutic strategies.

## Conclusion

The developed combined prediction system achieves superior performance in anticipating severe (grade≥3) ARD in NPC patients receiving tomotherapy. This tool facilitates pre-therapeutic risk stratification, dosimetric parameter refinement, and evidence-based scheduling of preventive skin management protocols, offering a paradigm-shifting approach to individualized cutaneous protection strategies.

## Data Availability

The raw data supporting the conclusions of this article will be made available by the authors, without undue reservation.
