# Supplementary material for: Predictive modeling of acute radiation-induced dermatitis in nasopharyngeal carcinoma patients undergoing tomotherapy using machine learning with multimodal data integration
Source: Front Oncol. 2025 Oct 2;15:1601493. doi: 10.3389/fonc.2025.1601493 (PMC12527865; doi:10.3389/fonc.2025.1601493)
Supplement: Supplementary file 1 [file Table1.docx]

**sTable1.** Predictive performance of all clinical models.

| **M**odel Name | **Accuracy** | **AUC** | **95%Cl** | **Sensitivity** | **Specificity** | **PPV** | **NPV** | **Precision** | **Recall** | **F1** |
| --- | --- | --- | --- | --- | --- | --- | --- | --- | --- | --- |
| LR |  |  |  |  |  |  |  |  |  |  |
| Train Cohort | 0.674 | 0.671 | 0.5756-0.7664 | 0.500 | 0.765 | 0.524 | 0.747 | 0.524 | 0.500 | 0.512 |
| Test Cohort | 0.688 | 0.747 | 0.5624-0.9311 | 0.636 | 0.714 | 0.538 | 0.789 | 0.538 | 0.636 | 0.583 |
| SVM |  |  |  |  |  |  |  |  |  |  |
| Train Cohort | 0.674 | 0.660 | 0.5600-0.7595 | 0.591 | 0.718 | 0.520 | 0.772 | 0.520 | 0.591 | 0.553 |
| Test Cohort | 0.719 | 0.721 | 0.5227-0.9189 | 0.455 | 0.857 | 0.625 | 0.750 | 0.625 | 0.455 | 0.526 |
| KNN |  |  |  |  |  |  |  |  |  |  |
| Train Cohort | 0.705 | 0.774 | 0.6982-0.8496 | 0.341 | 0.894 | 0.625 | 0.724 | 0.625 | 0.341 | 0.441 |
| Test Cohort | 0.500 | 0.515 | 0.3161-0.7142 | 0.455 | 0.524 | 0.333 | 0.647 | 0.333 | 0.455 | 0.385 |
| RandomForest |  |  |  |  |  |  |  |  |  |  |
| Train Cohort | 0.798 | 0.895 | 0.8432-0.9470 | 0.909 | 0.741 | 0.645 | 0.940 | 0.645 | 0.909 | 0.755 |
| Test Cohort | 0.438 | 0.387 | 0.1908-0.5841 | 0.636 | 0.333 | 0.333 | 0.636 | 0.333 | 0.636 | 0.437 |
| ExtraTrees |  |  |  |  |  |  |  |  |  |  |
| Train Cohort | 0.822 | 0.925 | 0.8847-0.9647 | 0.591 | 0.941 | 0.839 | 0.816 | 0.839 | 0.591 | 0.693 |
| Test Cohort | 0.656 | 0.377 | 0.1777-0.5756 | 0.000 | 1.000 | 0.000 | 0.656 | 0.000 | 0.000 | NaN |
| XGBoost |  |  |  |  |  |  |  |  |  |  |
| Train Cohort | 0.713 | 0.830 | 0.7603-0.9002 | 0.841 | 0.647 | 0.552 | 0.887 | 0.552 | 0.841 | 0.667 |
| Test Cohort | 0.531 | 0.615 | 0.4149-0.8146 | 0.636 | 0.476 | 0.389 | 0.714 | 0.389 | 0.636 | 0.483 |
| LightGBM |  |  |  |  |  |  |  |  |  |  |
| Train Cohort | 0.643 | 0.704 | 0.6133-0.7955 | 0.636 | 0.647 | 0.483 | 0.775 | 0.483 | 0.636 | 0.549 |
| Test Cohort | 0.625 | 0.753 | 0.5801-0.9264 | 0.818 | 0.524 | 0.474 | 0.846 | 0.474 | 0.818 | 0.600 |
| MLP |  |  |  |  |  |  |  |  |  |  |
| Train Cohort | 0.643 | 0.406 | 0.2998-0.5131 | 0.068 | 0.941 | 0.375 | 0.661 | 0.375 | 0.068 | 0.115 |
| Test Cohort | 0.625 | 0.418 | 0.1943-0.6412 | 0.910 | 0.905 | 0.333 | 0.655 | 0.333 | 0.091 | 0.143 |

AUC, area under the receiver operating characteristic curve. KNN, K-Nearest Neighbors. LightGBM, Light Gradient Boosting Machine. LR, Logistic Regression. MLP, Multilayer Perceptron. NPV, Negative Predictive Value. PPV, Positive Predictive Value. SVM, Support Vector Machine. XGboost: eXtreme Gradient Boosting.
