# Supplementary material for: Predictive modeling of acute radiation-induced dermatitis in nasopharyngeal carcinoma patients undergoing tomotherapy using machine learning with multimodal data integration
Source: Front Oncol. 2025 Oct 2;15:1601493. doi: 10.3389/fonc.2025.1601493 (PMC12527865; doi:10.3389/fonc.2025.1601493)
Supplement: Supplementary file 2 [file Table2.docx]

**sTable2.** Predictive performance of all radiomics models.

| **M**odel Name | **Accuracy** | **AUC** | **95%Cl** | **Sensitivity** | **Specificity** | **PPV** | **NPV** | **Precision** | **Recall** | **F1** |
| --- | --- | --- | --- | --- | --- | --- | --- | --- | --- | --- |
| LR |  |  |  |  |  |  |  |  |  |  |
| Train Cohort | 0.736 | 0.822 | 0.7501-0.8938 | 0.727 | 0.741 | 0.593 | 0.840 | 0.593 | 0.727 | 0.653 |
| Test Cohort | 0.688 | 0.680 | 0.4811-0.8782 | 0.455 | 0.810 | 0.556 | 0.739 | 0.556 | 0.455 | 0.500 |
| SVM |  |  |  |  |  |  |  |  |  |  |
| Train Cohort | 0.760 | 0.865 | 0.7947-0.9353 | 0.932 | 0.671 | 0.594 | 0.950 | 0.594 | 0.932 | 0.726 |
| Test Cohort | 0.750 | 0.779 | 0.6040-0.9544 | 0.727 | 0.762 | 0.615 | 0.842 | 0.615 | 0.727 | 0.667 |
| KNN |  |  |  |  |  |  |  |  |  |  |
| Train Cohort | 0.736 | 0.797 | 0.7243-0.8696 | 0.432 | 0.894 | 0.679 | 0.752 | 0.679 | 0.432 | 0.528 |
| Test Cohort | 0.719 | 0.660 | 0.4568-0.8636 | 0.182 | 1.000 | 1.000 | 0.700 | 1.000 | 0.182 | 0.308 |
| RandomForest |  |  |  |  |  |  |  |  |  |  |
| Train Cohort | 0.969 | 1.000 | 1.0000-1.0000 | 0.909 | 1.000 | 1.000 | 0.955 | 1.000 | 0.909 | 0.952 |
| Test Cohort | 0.688 | 0.608 | 0.3746-0.8418 | 0.182 | 0.952 | 0.667 | 0.690 | 0.667 | 0.182 | 0.286 |
| ExtraTrees |  |  |  |  |  |  |  |  |  |  |
| Train Cohort | 0.659 | 1.000 | 1.0000-1.0000 | 0.000 | 1.000 | 0.000 | 0.659 | 0.000 | 0.000 | NaN |
| Test Cohort | 0.656 | 0.571 | 0.3393-0.8035 | 0.091 | 0.952 | 0.500 | 0.667 | 0.500 | 0.091 | 0.154 |
| XGBoost |  |  |  |  |  |  |  |  |  |  |
| Train Cohort | 0.992 | 1.000 | 1.0000-1.0000 | 0.977 | 1.000 | 1.000 | 0.988 | 1.000 | 0.977 | 0.989 |
| Test Cohort | 0.656 | 0.632 | 0.4113-0.8528 | 0.545 | 0.714 | 0.500 | 0.750 | 0.500 | 0.545 | 0.522 |
| LightGBM |  |  |  |  |  |  |  |  |  |  |
| Train Cohort | 0.868 | 0.908 | 0.8527-0.9628 | 0.818 | 0.894 | 0.800 | 0.905 | 0.800 | 0.818 | 0.809 |
| Test Cohort | 0.750 | 0.699 | 0.4674-0.9308 | 0.636 | 0.810 | 0.636 | 0.810 | 0.636 | 0.636 | 0.636 |
| MLP |  |  |  |  |  |  |  |  |  |  |
| Train Cohort | 0.682 | 0.755 | 0.6701-0.8406 | 0.750 | 0.647 | 0.524 | 0.833 | 0.524 | 0.750 | 0.617 |
| Test Cohort | 0.469 | 0.519 | 0.3078-0.7312 | 0.818 | 0.286 | 0.375 | 0.750 | 0.375 | 0.818 | 0.514 |

AUC, area under the receiver operating characteristic curve. KNN, K-Nearest Neighbors. LightGBM, Light Gradient Boosting Machine. LR, Logistic Regression. MLP, Multilayer Perceptron. NPV, Negative Predictive Value. PPV, Positive Predictive Value. SVM, Support Vector Machine. XGboost: eXtreme Gradient Boosting.
