# Supplementary material for: Predictive modeling of acute radiation-induced dermatitis in nasopharyngeal carcinoma patients undergoing tomotherapy using machine learning with multimodal data integration
Source: Front Oncol. 2025 Oct 2;15:1601493. doi: 10.3389/fonc.2025.1601493 (PMC12527865; doi:10.3389/fonc.2025.1601493)
Supplement: Supplementary file 3 [file Table3.docx]

**sTable3.** Predictive performance of all dosomics models.

| **M**odel Name | **Accuracy** | **AUC** | **95%Cl** | **Sensitivity** | **Specificity** | **PPV** | **NPV** | **Precision** | **Recall** | **F1** |
| --- | --- | --- | --- | --- | --- | --- | --- | --- | --- | --- |
| LR |  |  |  |  |  |  |  |  |  |  |
| Train Cohort | 0.574 | 0.640 | 0.5401-0.7401 | 0.795 | 0.459 | 0.432 | 0.812 | 0.432 | 0.795 | 0.560 |
| Test Cohort | 0.656 | 0.641 | 0.4206-0.8608 | 0.636 | 0.667 | 0.500 | 0.778 | 0.500 | 0.636 | 0.560 |
| SVM |  |  |  |  |  |  |  |  |  |  |
| Train Cohort | 0.674 | 0.649 | 0.5466-0.7512 | 0.545 | 0.741 | 0.522 | 0.759 | 0.522 | 0.545 | 0.533 |
| Test Cohort | 0.656 | 0.632 | 0.4193-0.8447 | 0.636 | 0.667 | 0.500 | 0.778 | 0.500 | 0.636 | 0.560 |
| KNN |  |  |  |  |  |  |  |  |  |  |
| Train Cohort | 0.682 | 0.803 | 0.7293-0.8765 | 0.068 | 1.000 | 1.000 | 0.675 | 1.000 | 0.068 | 0.128 |
| Test Cohort | 0.656 | 0.463 | 0.2455-0.6809 | 0.000 | 1.000 | 0.000 | 0.656 | 0.000 | 0.000 | NaN |
| RandomForest |  |  |  |  |  |  |  |  |  |  |
| Train Cohort | 0.938 | 0.997 | 0.9919-1.0000 | 0.841 | 0.988 | 0.974 | 0.923 | 0.974 | 0.841 | 0.902 |
| Test Cohort | 0.656 | 0.494 | 0.2525-0.7345 | 0.000 | 1.000 | 0.000 | 0.656 | 0.000 | 0.000 | NaN |
| ExtraTrees |  |  |  |  |  |  |  |  |  |  |
| Train Cohort | 0.659 | 1.000 | 1.0000-1.0000 | 0.000 | 1.000 | 0.000 | 0.659 | 0.000 | 0.000 | NaN |
| Test Cohort | 0.344 | 0.429 | 0.2227-0.6344 | 0.364 | 0.333 | 0.222 | 0.500 | 0.222 | 0.364 | 0.276 |
| XGBoost |  |  |  |  |  |  |  |  |  |  |
| Train Cohort | 0.814 | 0.884 | 0.8260-0.9414 | 0.818 | 0.812 | 0.692 | 0.896 | 0.692 | 0.818 | 0.750 |
| Test Cohort | 0.344 | 0.416 | 0.2050-0.6262 | 0.818 | 0.095 | 0.321 | 0.500 | 0.321 | 0.818 | 0.462 |
| LightGBM |  |  |  |  |  |  |  |  |  |  |
| Train Cohort | 0.659 | 0.702 | 0.6116-0.7919 | 0.545 | 0.718 | 0.500 | 0.753 | 0.500 | 0.545 | 0.522 |
| Test Cohort | 0.656 | 0.597 | 0.3653-0.8295 | 0.364 | 0.810 | 0.500 | 0.708 | 0.500 | 0.364 | 0.421 |
| MLP |  |  |  |  |  |  |  |  |  |  |
| Train Cohort | 0.574 | 0.640 | 0.5401-0.7401 | 0.795 | 0.459 | 0.432 | 0.812 | 0.432 | 0.795 | 0.560 |
| Test Cohort | 0.656 | 0.641 | 0.4206-0.8608 | 0.636 | 0.667 | 0.500 | 0.778 | 0.500 | 0.636 | 0.560 |

AUC, area under the receiver operating characteristic curve. KNN, K-Nearest Neighbors. LightGBM, Light Gradient Boosting Machine. LR, Logistic Regression. MLP, Multilayer Perceptron. NPV, Negative Predictive Value. PPV, Positive Predictive Value.SVM, Support Vector Machine. XGboost: eXtreme Gradient Boosting.
